# High resolution slice-selective Fourier Velocity Encoding using spiral SENSE with velocity unwrap

**DOI:** 10.1186/1532-429X-14-S1-O40

**Published:** 2012-02-01

**Authors:** Jennifer A Steeden, David Atkinson, Alexander Jones, Vivek Muthurangu

**Affiliations:** 1 Institude of Cardiovascular Science, UCL, London, UK; 2 Centre for Medical Imaging, UCL, London, UK

## Background

In patients with congenital heart disease (CHD), it is desirable to accurately measure peak velocity (Vmax). Unfortunately, phase-contrast MR (PCMR) tends to underestimate peak velocities. Fourier Velocity Encoding (FVE) can measure peak velocities in MRI, but is not commonly used due to long acquisition times.

Therefore, we have developed a FVE sequence that combines spiral trajectories with parallel imaging (SENSE), partial-Fourier acquisition and a novel velocity-unwrap technique. The aim of this study is to validate this sequence.

## Methods

FVE sequence: FVE was performed using a spiral trajectory (table 1). Spiral interleaves were undersampled (R=4) and reconstructed using an iterative SENSE algorithm. Partial Fourier (67%) was performed in kv with a homodyne reconstruction was used. The velocity-unwrap method purposefully aliases data in v (by acquiring half the number of kv-positions), and unwrapped using prior information about the flow direction. Peak velocity was determined using previously described techniques 1-3.

**Table 1:** Imaging Parameters

	lr-PCMR	hr-PCMR	FVE
TE/TR (ms)	~2.2/5.0	~2.2/5.0	~3.5/10.3
Readouts	Cartesian	Cartesian	Spiral: 16 interleaves
Acceleration factor (in kx-ky)	2 (GRAPPA)	2 (GRAPPA)	4 (SENSE)
Matrix Size	128	256	192
Image FOV (mm)	320	320	450
Total Scan Duration (heartbeats)	15	108	15
Spatial Resolution (mm)	~2.5	~1.3	~2.3
Temporal Resolution (ms)	~40	~30	~41
Velocity Resolution (cm/s)	-	-	15-38

In-vitro: A pulsatile flow pump was connected to a tube phantom (13mm diameter) with a stenosis (6mm diameter). Peak velocity measurements using the following techniques were compared at 15 different flow rates; 1) US doppler, 2) low-resolution PCMR (lr-PCMR), 3) high-resolution PCMR (hr-PCMR), 4) FVE.

In-vivo: 12 CHD patients (7M:5F; 34.3±18.8 years) with stenoses were assessed. Peak velocity measurements were compared between; 1) lr-PCMR, 2) hr-PCMR, and 3) FVE.

## Results

In-vitro: There were no statistically significant differences between Vmax measured using US and FVE (table 2). However both PCMR sequences showed a statistically significant underestimation of peak flow compared to US (table 2). This is particularly true of lr-PCMR, which underestimated Vmax by >0.5m/s. In-vivo: There was a significant underestimation of Vmax measured using both PCMR sequences when compared to FVE (lr-PCMR; 229±42cm/s, hr-PCMR; 238±46cm/s, FVE; 256±67cm/s).

**Table 2:** In-vitro results

	Echo	lh-PCMR	hr-PCMR	FVE
Peak velocity (cm/s)	441±144	375±133^	398±136^	443±144
Bias* (cm/s)	-	-66	-42	+3
Limits of Agreement* (cm/s)	-	-26 to -105	-10 to -75	+17 to -12
Correlation coefficient* (r)	-	0.9926	0.9949	0.9987

* Calculated with echo ^ Value is significantly different (ANOVA) from echo (P < 0.05)

## Conclusions

Fourier velocity encoding allows accurate assessment of peak velocities as it measures a velocity spectrum per pixel, rather than the average velocity. However this extra encoding takes time, which has reduced its clinical effectiveness. We have shown that it possible to achieve high resolution FVE within a short breath-hold by combining spiral trajectories, parallel imaging, partial Fourier and velocity-unwrap. This sequence was shown to be significantly more accurate than PCMR in-vitro, and also to provide higher peak velocities than PCMR in-vivo. Thus, the sequence should be able to replace Doppler echocardiography making CMR a true one-stop-shop in assessing congenital heart disease.

## Funding

JAS: EPSRC PhD+.

VM: BHF.
